# Müllerian Cyst: An Uncommon Etiology of Chronic Pelvic Pain Syndrome in Men

**DOI:** 10.7759/cureus.49046

**Published:** 2023-11-19

**Authors:** Jihad Lakssir, Hicham El Boté, Omar Bellouki, Abdelmounim Boughaleb

**Affiliations:** 1 Urology A, Ibn Sina Hospital, Rabat, MAR; 2 Anatomy - Urology, Sultan Moulay Slimane University, Faculty of Medicine and Pharmacy, Beni Mellal, MAR

**Keywords:** chronic prostatitis, pain managment, chronic pelvic pain syndrome, intra-prostatic cyst, mullerien cyst

## Abstract

Chronic pelvic pain syndrome (CPPS) in men is a complex pathological entity with a delicate nosological diagnosis and multiple etiological hypotheses dominated by urological causes.

The Müllerian cyst has an embryological origin and is part of an organic anomaly of the male urogenital tract, incidentally detected during an initial infertility examination or in the presence of non-specific urinary symptoms.

By its mass effect, it puts tension on the pelvic floor muscles and induces a stimulation of nerves which could explain its implication in the CPPS.

Through this case report and literature review, we will clarify the etiopathogenesis and the diagnosis of CPPS as well as the approach to follow for better therapeutic management.

## Introduction

Chronic pelvic pain syndrome (CPPS) is a condition primarily affecting young men from 35 to 50 years of age. It is characterized by chronic pelvic pain associated with non-specific genitourinary signs. CPPS is a medical condition less common in men than in women and therefore it received less attention in urology.

There are several causes and forms of chronic pelvic pain in men, often mistaken for chronic prostatitis, providing the urologist with diagnostic challenges and eventually inappropriate treatments.

The Müllerian cyst, a rare entity typically benign intra-prostatic cyst, most often congenital with variable symptomatology, can lead to vas deferens obstruction through extrinsic compression. It is characterized by a large size, which increases pressure at the pelvic level, thus putting tension on the pelvic floor muscles [1].

 It is this increased pressure that would consequently cause pelvic pain through nerve compression.

Through a review of the literature and the analysis of the clinical observation of our young patient diagnosed with Müllerian cyst following CPPS, we will try to establish a causal link between these two pathological entities.

## Case presentation

Mr. A.E., aged 48 and a father of two children, has a medical history of well-managed insulin-dependent diabetes. He consulted for chronic perineal pain persisting for over six months despite a well-conducted analgesic treatment (Category II Analgesics, as per the WHO classification).

The interrogation revealed signs of the lower urinary tract including burning during urination, dysuria, and moderate pain during ejaculation.

**Figure 1:**
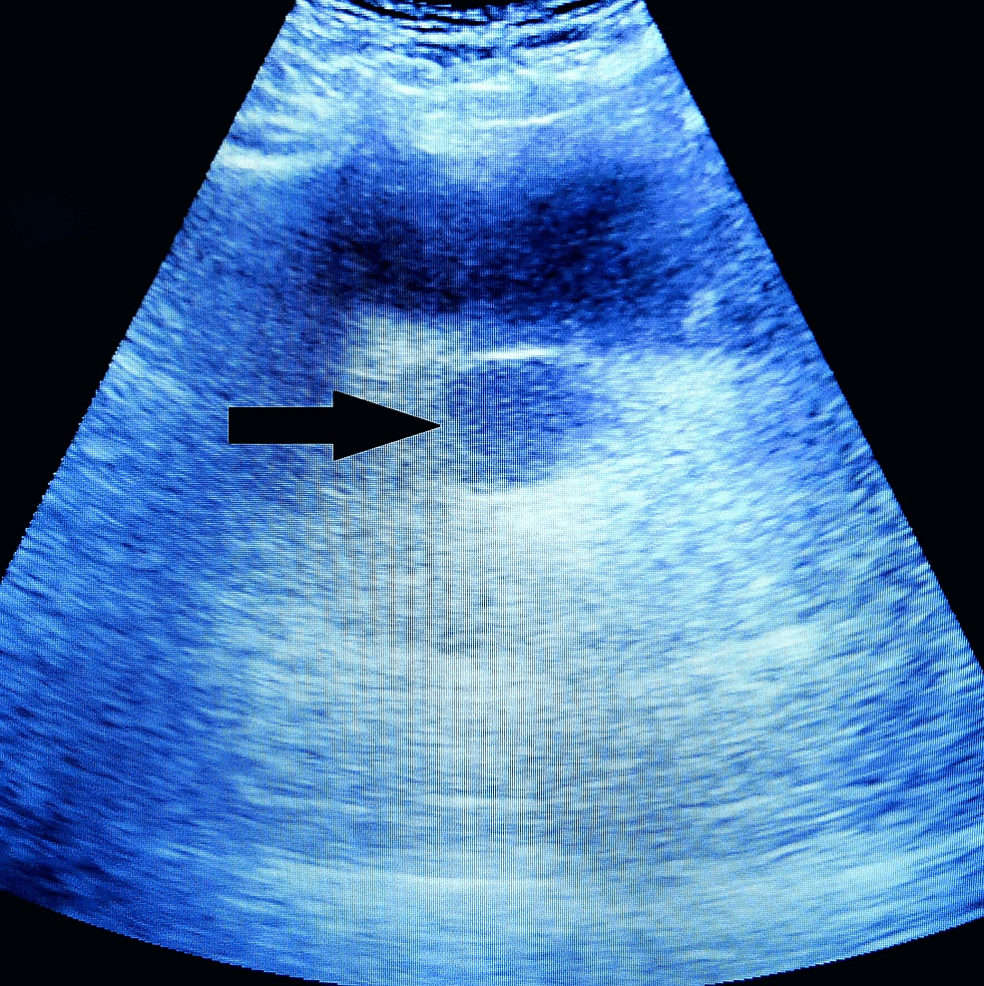
Image of a suprapubic ultrasound showing an intra-prostatic cyst.

The clinical assessment showed a soft, painless prostate and a palpable firm mass that appears anechoic on ultrasound examination (Figure 1). Meanwhile, the pelvic neurological examination revealed no abnormalities.

**Figure 2:**
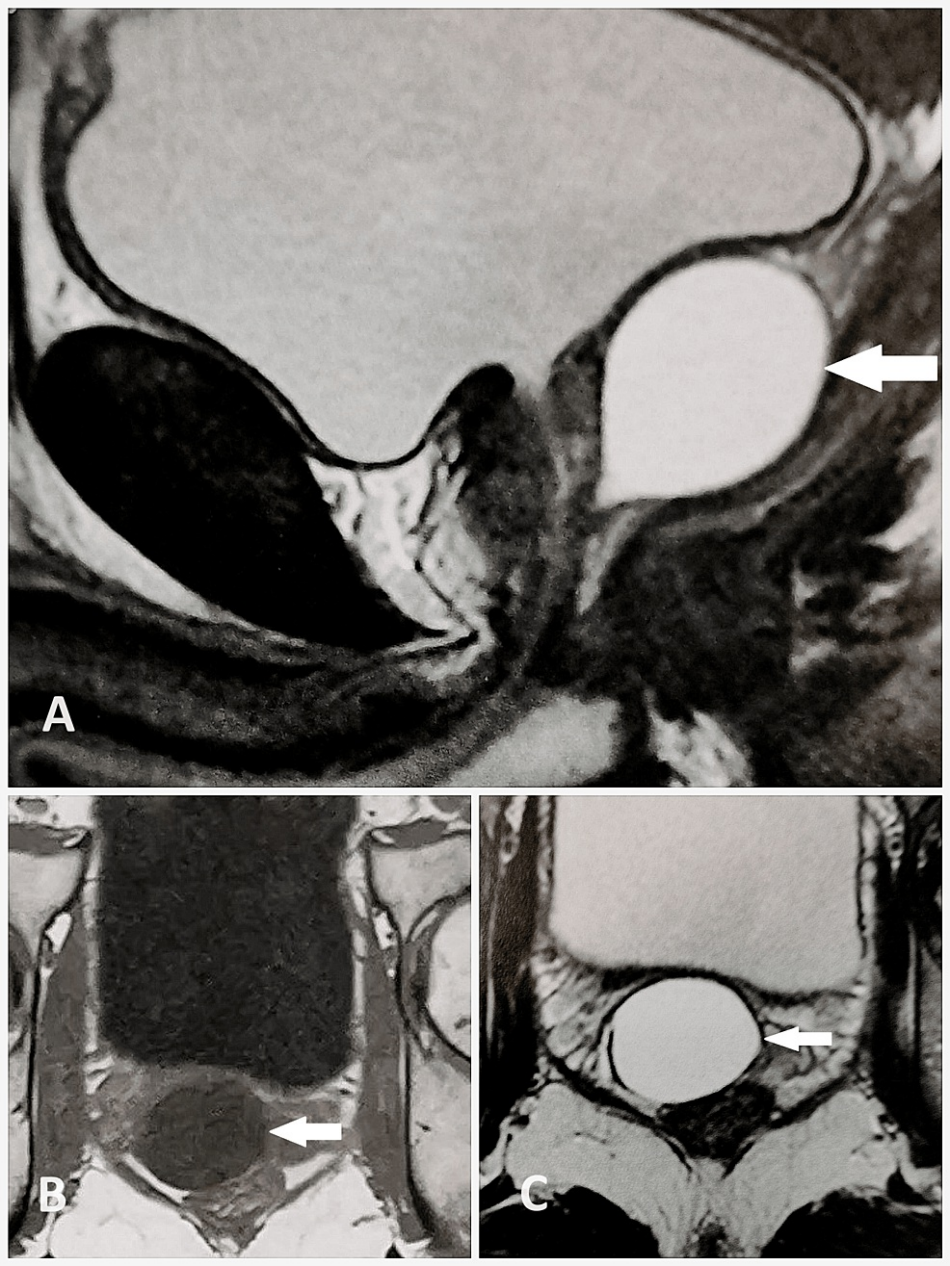
MRI image showing a round cystic mass in the prostate. A: Sagittal view of the T2-weighted sequence. B: Axial view of the T1-weighted sequence. C: Axial view of the T2-weighted sequence.

Pelvic MRI confirmed the presence of an intra-prostatic cyst, hyperintense on T2-weighted sequence and hypointense T1-weighted sequence, not enhanced by gadolinium injection, likely of Müllerian origin, measuring 38x28x32 mm (Figure 2).

In addition, the cytobacteriological examination of the urine, the sperm culture, and the urethral swab were sterile except for a significant leukocyturia.

**Figure 3:**
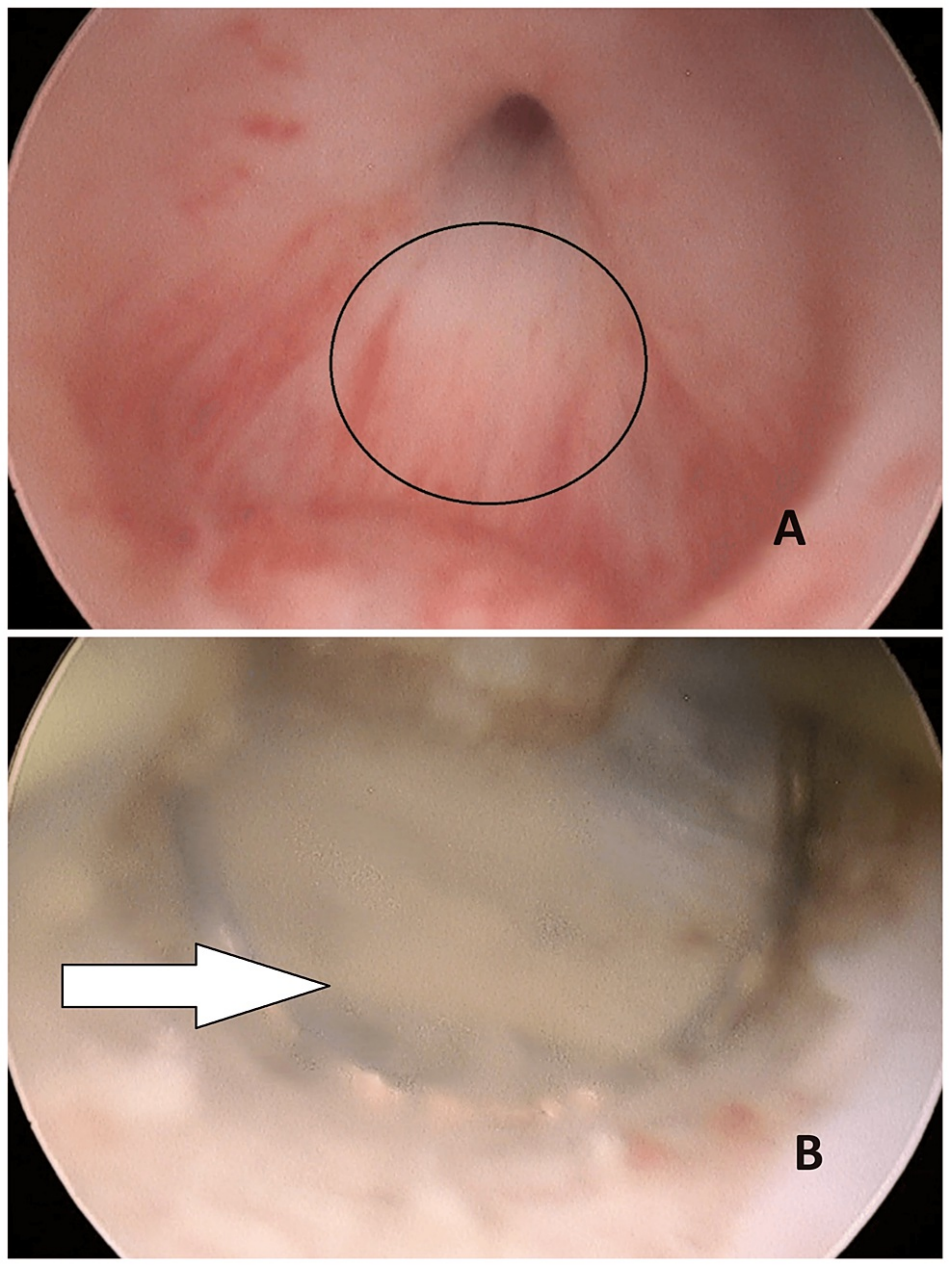
Image showing the endoscopic aspect of the Müllerian cyst. A: The wall of Müllerian cyst (before resection). B: Evacuation of a yellowish and viscous liquid.

After informing the patient, an endoscopic resection of the anterior wall of the cyst was performed, while preserving the bladder neck and the external sphincter, leading to an evacuation of a yellowish and viscous liquid (Figure 3).

Anatomopathological examination confirmed the Müllerian cyst’s nature.

Three months postoperatively, the patient showed favorable evolution with the disappearance of urinary signs.

## Discussion

CPPS is less frequently observed in males compared to females, lasting for over six months, and is often mistakenly diagnosed as a variant of chronic prostatitis. However, according to the National Institute of Health (NIH) [2], this syndrome is classified as type III, distinguishing it from acute and chronic bacterial prostatitis.

The comprehension of this syndrome frequently implies an underlying neurological basis, as explained by Zerman et al. [3]. A minor noxious stimulus (such as prostatitis or an intra-prostatic cyst) affects the complex and interactive innervation of the pelvic organs by modifying and resetting the effector pathways (concept of neuronal plasticity).

The repetitive painful stimulus leads to an amplified and disproportionate modification of the interactive and reflex coordination resulting in pathological neuronal changes.

The urological diagnostic work-up requires a thorough history with particular emphasis on micturition, defecation, erection, and ejaculation. For an objective assessment of the primary symptom, pain, it is advisable to employ a Visual Analog Pain Scale (VAS) along with a voiding diary. Also, the clinical examination should focus on rectal palpation for evaluating the condition of the prostate, rectum, anal sphincter, as well as the sensitivity and tone of the pelvic floor muscles [4].

The diagnosis of CPPS relies on the absence of bacterial infection markers in the cytobacteriological examination of urine, and the presence of hyperleukocytosis that indicates an inflammatory origin [3].

As the diagnostic process advances, the underlying pathology is unveiled. In our case, a Müllerian cyst was suspected through the results of the clinical examination and the CBEU (cytobacteriological examination of urine) results, later confirmed by radiological imaging displaying an intra-prostatic cyst.

Also, the Müllerian cyst is characterized by oligo or azoospermia with a low volume of ejaculate as observed in the spermogram, and the presence of a yellowish liquid with an absence of spermatozoa [1] during ultrasound-guided puncture aspiration. In addition, MRI is becoming an important tool in prostatic exploration, particularly in cystic pathology.

The management of such cases is often challenging and involves multiple facets, including the treatment of CPPS and the presumed underlying pathology.

In the case of Müllerian cysts, several techniques have been described, starting with minimally invasive techniques, such as puncture aspiration, though these are associated with a high recurrence rate. Secondly, endoscopic resection of the cyst’s anterior wall has shown favorable outcomes [5]. Finally, a more invasive surgical treatment, such as the extra bladder approach, which exposes the patient to the risk of adjacent elements' injury at the bladder neck, or the trans-vesical approach, which exposes the patient to extended bladder catheterization.

The treatment of CPPS is more complicated due to the patient's history of multiple unsuccessful treatments. Initially, a multidisciplinary approach is adopted with the aim of regaining patient trust and minimizing medication usage. It is founded on three essential points, starting an effective analgesic treatment to be instituted in parallel with the etiological investigation, engaging in perineal reeducation to attain complete relaxation of the pelvic musculature, and employing drugs to potentiate analgesic effects and aid in pelvic relaxation such as the alpha-blockers. The use of benzodiazepines can also be effective due to their influence on spinal muscle tone regulation and their central sedative effect [4].

## Conclusions

Our understanding of the physiopathology of CPPS has historically been imprecise, leading to numerous therapeutic challenges. The neuropsychological mechanism seems to play a prominent role.

The interplay of pain with urinary signs, a decline in quality of life, and a psychological impact highlight the necessity for a combination of several therapies along with regular re-evaluation and comprehensive, multidisciplinary management.
